# Causal association between gut microbiota and intrahepatic cholestasis of pregnancy: mendelian randomization study

**DOI:** 10.1186/s12884-023-05889-8

**Published:** 2023-08-05

**Authors:** Chuang Li, Na Li, Caixia Liu, Shaowei Yin

**Affiliations:** 1 Department of Obstetrics & Gynecology, Shengjing Hospital of China Medical University, Shenyang, Liaoning Province 110004 China; 2 Liaoning Key Laboratory of Maternal-Fetal Medicine, Shengjing Hospital of China Medical University, Shenyang, Liaoning Province 110004 China

**Keywords:** Intrahepatic cholestasis of pregnancy, Gut microbiota, Causal relationship, Mendelian randomization, Single nucleotide polymorphism, Instrumental variable

## Abstract

**Background:**

Previous observational cohort studies have shown that the composition of the gut microbiota is related to the risk of intrahepatic cholestasis of pregnancy (ICP), although it is unclear if the association is causative. This study used Mendelian randomization (MR) to systematically examine whether the gut microbiota was causally linked to ICP.

**Methods:**

We obtained the genome-wide association study (GWAS) summary statistics of gut microbiota and ICP from published GWASs. Maximum likelihood (ML), MR-Egger regression, weighted median, inverse variance weighted (IVW), and weighted model were used to investigate the causal association between gut microbiota and ICP. We further conducted a series of sensitivity analyses to confirm the robustness of the primary results of the MR analyses. Reverse MR analysis was performed on the bacterial taxa that were reported to be causally linked to ICP risk in forwarding MR analysis to evaluate the possibility of reverse causation.

**Results:**

MR analysis revealed that phylum *Tenericutes* (OR: 1.670, 95%CI: 1.073–2.598, *P* = 0.023), class *Bacteroidia* (OR: 1.644, 95%CI: 1.031–2.622, *P* = 0.037), class *Mollicutes* (OR: 1.670, 95%CI: 1.073–2.598, *P* = 0.023), and order *Bacteroidales* (OR: 1.644, 95%CI: 1.031–2.622, *P* = 0.037), and were positively associated with the risk of ICP. And we identified that the relative abundance of genus *Dialister* (OR: 0.562, 95%CI: 0.323–0.977, *P* = 0.041), genus *Erysipelatoclostridium* (OR: 0.695, 95%CI: 0.490–0.987, *P* = 0.042), genus *Eubacterium* (*brachy group*) (OR: 0.661, 95%CI: 0.497–0.880, *P* = 0.005), genus *Eubacterium* (*hallii group*) (OR: 0.664, 95%CI: 0.451–0.977, *P* = 0.037), genus *Holdemania* (OR: 0.590, 95%CI: 0.414–0.840, *P* = 0.003), genus *Ruminococcus* (*torques group*) (OR: 0.448, 95%CI: 0.235–0.854, *P* = 0.015), and genus *Veillonella* (OR: 0.513, 95%CI: 0.294–0.893, *P* = 0.018) were related to a lower risk of ICP. Additional sensitivity analyses confirmed the robustness of the association between specific gut microbiota composition and ICP. No evidence of reverse causality from ICP to identified bacterial taxa was found in the findings of the reverse MR analyses.

**Conclusions:**

Under MR assumptions, our findings propose new evidence of the relationship between gut microbiota and ICP risk. Our results show that the gut microbiota may be useful target of intervention for ICP.

**Supplementary Information:**

The online version contains supplementary material available at 10.1186/s12884-023-05889-8.

## Introduction

Intrahepatic cholestasis of pregnancy (ICP), typically presenting in the 2nd or 3rd trimester, is the most common liver disease specific to pregnancy [1]. ICP affects 0.3–5.6% of pregnant women, with marked differences by ethnicity [2]. ICP is characterized by the new-onset maternal pruritus and elevated serum bile acids concentration, and maternal symptoms and biochemical test abnormalities resolve after delivery [1, 3, 4]. Numerous studies revealed that the development of ICP is characterized by a decrease in bile flow through the liver and subsequent excretion, which would finally result in an intrahepatic accumulation of toxic bile acids [5]. ICP has impacts on both maternal and fetal health, with the effects on the fetus being the most concerning. Increased bile acid peak concentrations are associated with multiple adverse perinatal outcomes, including meconium-stained amniotic fluid, spontaneous preterm birth, stillbirth, fetal asphyxia, and neonatal unit admission [2, 6, 7]. The dangers of ICP call for more investigation into the causes of this condition.

Growing evidences have indicated the observational associations between gut microbiota and the metabolism of bile acids. Gut microbiota is involved in multi biological processes that contribute to the metabolism of bile acids. Sayin et al. demonstrated that gut microbiota played a vital role in the metabolism of deconjugated primary bile acids into secondary bile acids through a series of enzymatic reactions [8]. Gut microbiota could also regulate bile acid synthesis in the liver by alleviating farnesoid X receptor (FXR) inhibition in the ileum [8]. An animal study also discovered the probiotic could improve bile acid metabolism dysregulation in pregnant rats [9]. But the studies investigating the relationships between gut microbiota and ICP are largely of observational nature. Conventional observational studies have bias and confounding factors that make it difficult to draw firm conclusions about whether or not a particular bacterial taxon is significantly linked to the risk of ICP [10].

Mendelian randomization (MR) is an approach integrating summary data of genome-wide association studies (GWAS) to estimate the causal link between risk factors and outcomes [11]. Genetic polymorphisms that are randomly assigned at conception are used in MR design as instrumental variables, which can fill in the gaps in the evidence by reducing confounding variables [12]. MR method must conform to three important assumptions. First, the instrumental variables must be associated with the risk factors of interest. Second, instrumental variables must be independent of confounders that influence risk factors and outcomes. Third, the instrumental variables can only influence the outcome through the risk factor [13, 14].

Thus, to determine the causal effect of gut microbiota on ICP risk, we carried out a bidirectional two sample MR design for the first time. We selected genetic variants significantly associated with specific intestinal flora as instrumental variables (IVs) to improve interference for a possible influence of gut microbiota on ICP.

## Methods

### Data sources

**Fig. 1:**
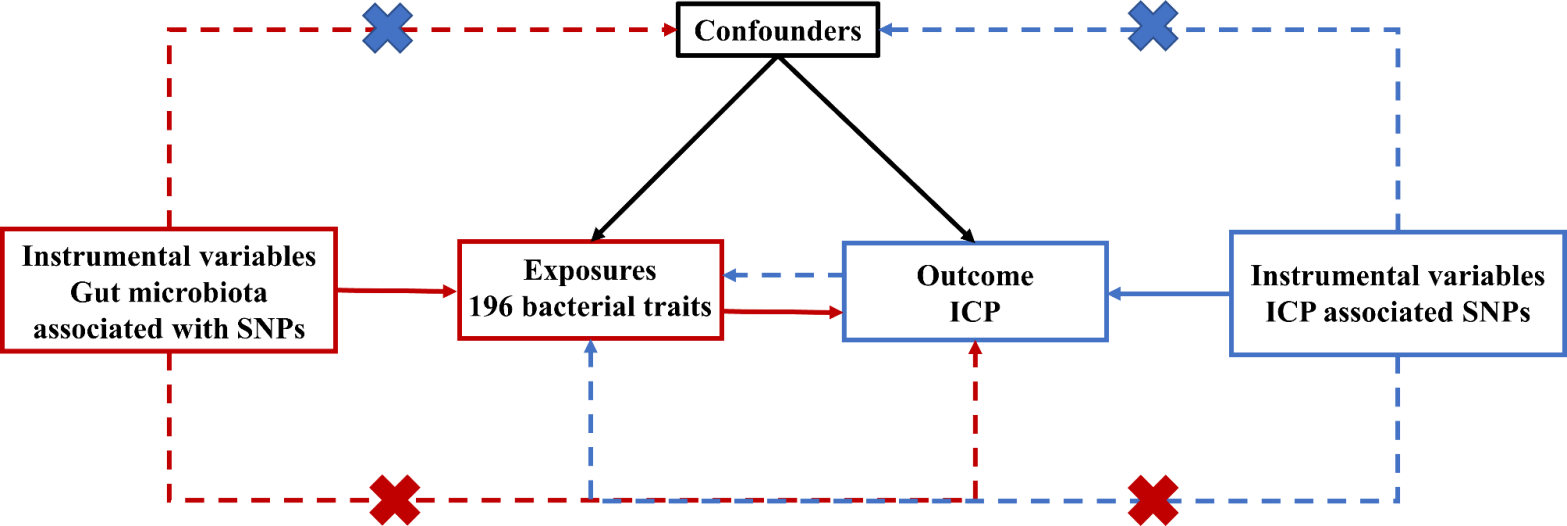
Study design of the bidirectional MR study of the associations between gut microbiota and ICP

Summary-level data for human gut microbiota were collected from a GWAS meta-analysis published to date for gut microbiota composition performed by the MiBioGen consortium [15, 16]. To investigate how human genetics affect the gut microbiota, the study coordinated the 16 S rRNA gene sequencing profiles and genome-wide genotypes of 18,340 individuals from 24 cohorts, the majority of whom were of European ancestry [16]. In the study, we excluded the 15 bacterial taxa without specific species names (unknown family or genus). As a result, 196 bacterial taxa (119 genera, 32 families, 20 orders, 16 classes, and 9 phyla) were included in the current study for analysis. FinnGen collaboration provided the GWAS summary data for ICP, which included 940 cases and 122,639 controls of European ancestry. A brief description of the bidirectional MR design is displayed in Fig. 1.

### Instrumental variable selection

The selection criteria for selecting optimal IVs were as follows. First, to obtain more comprehensive results, we selected a set of single nucleotide polymorphisms (SNPs) strongly associated with specific bacterial taxa (*p* < 1.0 × 10^–5^) as IVs [17]. Second, SNPs associated with each bacterial taxa were clumped to retain only independent SNPs. The linkage disequilibrium (LD) threshold for clumping was set to r^2^ < 0.001, with a clumping distance of 10,000 kb. LD was calculated based on the 1000 Genomes project European samples data reference panel. Third, the proxy SNPs (r^2^ > 0.8) would be selected to substitute the variants of interest, which were absent in the outcome summary statistic. Fourth, palindromic SNPs are removed from the chosen IVs in this MR analysis. Fifth, the strength of IVs was evaluated by calculating the *F*-statistic. If the corresponding F-statistic was much greater than 10, it was considered that there was small possibility of weak IVs bias [18].

### Statistical analysis

We applied fixed-effect inverse variance weighted (IVW) method for the primary MR analysis, with the random-effects modelling used in the presence of potential heterogeneity among selected SNPs [19]. The heterogeneity of IVs was measured using Cochran’s Q statistics. To derive a comprehensive evaluation of the impact of risk factors on outcomes, Maximum likelihood (ML) [20], Mendelian randomization Egger (MR Egger) regression [20], weighted median [21], and weighted model [21] were also conducted to infer the causality, although these methods have less statistical power than does the IVW test.

To evaluate the robustness of the detected causal effect of gut microbiota on ICP, we further carried out a series of sensitivity analyses, including the MR-Egger regression, Mendelian Randomization Pleiotropy RESidual Sum and Outlier (MR-PRESSO) analysis, and leave-one-out sensitivity analysis. The intercept from the MR-Egger could provide a formal test of directional pleiotropy [22]. The MR-PRESSO analysis detects the pleiotropic biases and corrects horizontal pleiotropy by removing the outliers [23]. Each instrumental SNP was removed one at a time during the leave-one-out sensitivity analysis to determine whether any possible SNPs with strong influences existed. In addition, reverse MR analyses were carried out to infer whether there was a reverse causal link between identified bacteria in forward MR analysis and ICP.

All statistical analyses were performed in R (version 4.1.3) with TwosampleMR [24, 25] and MR-PRESSO packages [23].

## Results

### Causal effect of gut microbiota on ICP

According to the selection criteria of IVs, a total of 2108 SNPs were used as IVs for 195 intestinal floras. The details about the used genetic variants in the MR analysis were shown in the Table S1. The *F*-statistic of the selected SNPs were all larger than 10, indicating the research was not susceptible to weak instrumental variables bias.

**Fig. 2:**
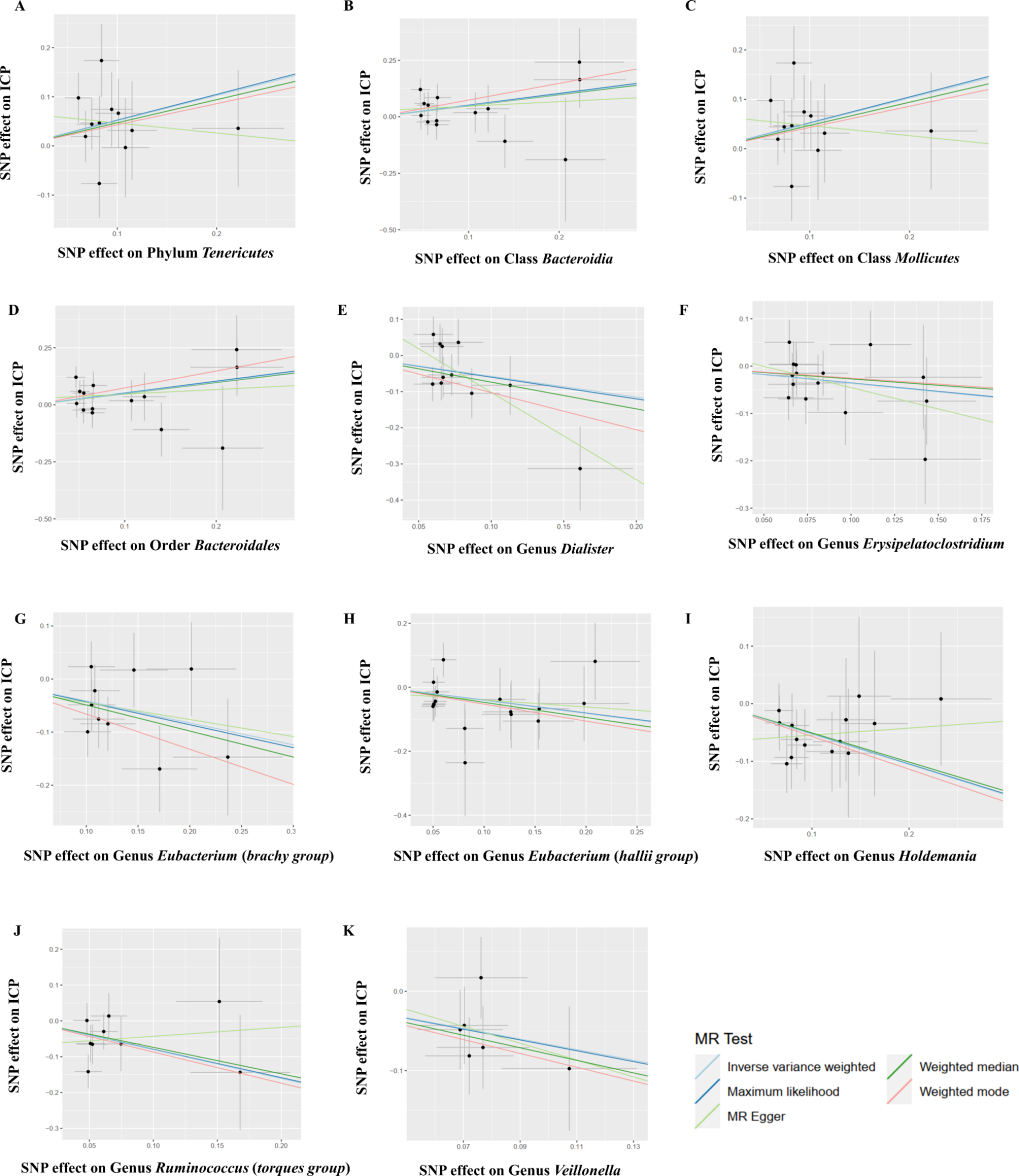
Scatter plots for the causal effect of gut microbiota on ICP. **(A)** Phylum *Tenericutes***(B)** Class Bacteroidia **(C)** Class *Mollicutes***(D)** Order *Bacteroidales***(E)** Genus *Dialister***(F)** Genus *Erysipelatoclostridium***(G)** Genus *Eubacterium* (*brachy group*) **(H)** Genus *Eubacterium* (*hallii group*) **(I)** Genus *Holdemania***(J)** Genus *Ruminococcus* (*torques group*) **(K)** Genus *Veillonella*. Plot showing the effect sizes of the SNP effects on ICP (y-axes) and the SNP effects on bacterial traits (x-axes)

As illustrated in Table 1; Figs. 2 and 11 bacterial taxa, including phylum *Tenericutes*, class *Bacteroidia*, class *Mollicutes*, order *Bacteroidales*, genus *Dialister*, genus *Erysipelatoclostridium*, genus *Eubacterium (brachy group)*, genus *Eubacterium (hallii group)*, genus *Holdemania*, genus *Ruminococcus (torques group)*, and genus *Veillonella* were associated with the risk of ICP in at least one MR method. Specifically, the results of IVW analysis showed that phylum *Tenericutes* (OR: 1.670, 95%CI: 1.073–2.598, *P* = 0.023), class *Bacteroidia* (OR: 1.644, 95%CI: 1.031–2.622, *P* = 0.037), class *Mollicutes* (OR: 1.670, 95%CI: 1.073–2.598, *P* = 0.023), order *Bacteroidales* (OR: 1.644, 95%CI: 1.031–2.622, *P* = 0.037), and were positively associated with the risk of ICP. The relative abundance of genus *Dialister* (OR: 0.562, 95%CI: 0.323–0.977, *P* = 0.041), genus *Erysipelatoclostridium* (OR: 0.695, 95%CI: 0.490–0.987, *P* = 0.042), genus *Eubacterium* (*brachy group*) (OR: 0.661, 95%CI: 0.497–0.880, *P* = 0.005), genus *Eubacterium* (*hallii group*) (OR: 0.664, 95%CI: 0.451–0.977, *P* = 0.037), genus *Holdemania* (OR: 0.590, 95%CI: 0.414–0.840, *P* = 0.003), genus *Ruminococcus* (*torques group*) (OR: 0.448, 95%CI: 0.235–0.854, *P* = 0.015), and genus *Veillonella* (OR: 0.513, 95%CI: 0.294–0.893, *P* = 0.018) were negatively related to the risk of ICP.

**Table 1:** MR estimates of causal effect of gut microbiota on ICP

Bacterial taxa (exposure)	MR method	No. SNP	Beta	SE	OR	95%CI	*P-value*
Phylum *Tenericutes*	ML	12	0.524	0.232	1.689	1.071–2.663	0.024*
	MR-Egger	12	-0.204	0.721	0.815	0.198–3.351	0.784
	Weighted median	12	0.470	0.305	1.601	0.880–2.911	0.123
	IVW	12	0.513	0.226	1.670	1.073–2.598	0.023*
	Weighted mode	12	0.430	0.407	1.537	0.692–3.411	0.316
Class *Bacteroidia*	ML	14	0.518	0.243	1.678	1.042–2.701	0.033*
	MR-Egger	14	0.200	0.487	1.222	0.470–3.175	0.688
	Weighted median	14	0.487	0.343	1.628	0.831–3.189	0.156
	IVW	14	0.497	0.238	1.644	1.031–2.622	0.037*
	Weighted mode	14	0.740	0.480	2.096	0.818–5.368	0.147
Class *Mollicutes*	ML	12	0.524	0.232	1.689	1.071–2.663	0.024*
	MR-Egger	12	-0.204	0.721	0.815	0.198–3.351	0.784
	Weighted median	12	0.470	0.302	1.601	0.886–2.892	0.119
	IVW	12	0.513	0.226	1.670	1.073–2.598	0.023*
	Weighted mode	12	0.430	0.424	1.537	0.670–3.525	0.335
Order *Bacteroidales*	ML	14	0.518	0.243	1.678	1.042–2.701	0.033*
	MR-Egger	14	0.200	0.487	1.222	0.470–3.175	0.688
	Weighted median	14	0.487	0.328	1.628	0.856–3.093	0.137
	IVW	14	0.497	0.238	1.644	1.031–2.622	0.037*
	Weighted mode	14	0.740	0.500	2.096	0.787–5.581	0.163
Genus *Dialister*	ML	11	-0.600	0.245	0.549	0.340–0.886	0.014*
	MR-Egger	11	-2.413	1.038	0.090	0.012–0.684	0.045*
	Weighted median	11	-0.739	0.360	0.478	0.236–0.977	0.040*
	IVW	11	-0.576	0.282	0.562	0.323–0.977	0.041*
	Weighted mode	11	-1.027	0.644	0.358	0.101–1.264	0.141
Genus *Erysipelatoclostridium*	ML	15	-0.356	0.183	0.701	0.490–1.002	0.051
	MR-Egger	15	-0.899	0.703	0.407	0.103–1.613	0.223
	Weighted median	15	-0.270	0.242	0.764	0.475–1.226	0.264
	IVW	15	-0.363	0.179	0.695	0.490–0.987	0.042*
	Weighted mode	15	-0.253	0.385	0.776	0.365–1.650	0.521
Genus *Eubacterium* (*brachy group*)	ML	10	-0.431	0.152	0.650	0.483–0.875	0.005*
	MR-Egger	10	-0.322	0.608	0.725	0.220–2.387	0.611
	Weighted median	10	-0.490	0.210	0.613	0.406–0.924	0.019*
	IVW	10	-0.413	0.146	0.661	0.497–0.880	0.005*
	Weighted mode	10	-0.662	0.371	0.516	0.249–1.067	0.108
Genus *Eubacterium* (*hallii group*)	ML	16	-0.403	0.202	0.668	0.450–0.993	0.046*
	MR-Egger	16	-0.217	0.406	0.805	0.363–1.785	0.601
	Weighted median	16	-0.472	0.265	0.624	0.371–1.049	0.075
	IVW	16	-0.410	0.197	0.664	0.451–0.977	0.037*
	Weighted mode	16	-0.528	0.362	0.590	0.290–1.198	0.165
Genus *Holdemania*	ML	14	-0.523	0.185	0.592	0.412–0.852	0.005*
	MR-Egger	14	0.121	0.532	1.129	0.398-3.200	0.823
	Weighted median	14	-0.507	0.249	0.602	0.370–0.981	0.042*
	IVW	14	-0.528	0.180	0.590	0.414–0.840	0.003*
	Weighted mode	14	-0.569	0.398	0.566	0.260–1.234	0.176
Genus *Ruminococcus* (*torques group*)	ML	9	-0.792	0.339	0.453	0.233–0.880	0.019*
	MR-Egger	9	0.250	1.044	1.284	0.166–9.937	0.818
	Weighted median	9	-0.739	0.443	0.478	0.200-1.139	0.096
	IVW	9	-0.804	0.329	0.448	0.235–0.854	0.015*
	Weighted mode	9	-0.869	0.614	0.419	0.126–1.397	0.195
Genus *Veillonella*	ML	6	-0.681	0.293	0.506	0.285–0.898	0.020*
	MR-Egger	6	-1.060	2.233	0.346	0.004–27.561	0.660
	Weighted median	6	-0.791	0.352	0.454	0.227–0.905	0.025*
	IVW	6	-0.668	0.283	0.513	0.294–0.893	0.018*
	Weighted mode	6	-0.870	0.492	0.419	0.160–1.100	0.137

MR, Mendelian randomization; ICP, Intrahepatic cholestasis of pregnancy; SNP, Single nucleotide polymorphism; OR, Odds ratio; CI, Confidence interval; ML, Maximum likelihood; IVW, Inverse variance weighted

*P-value < 0.05

### Sensitivity analysis

**Fig. 3:**
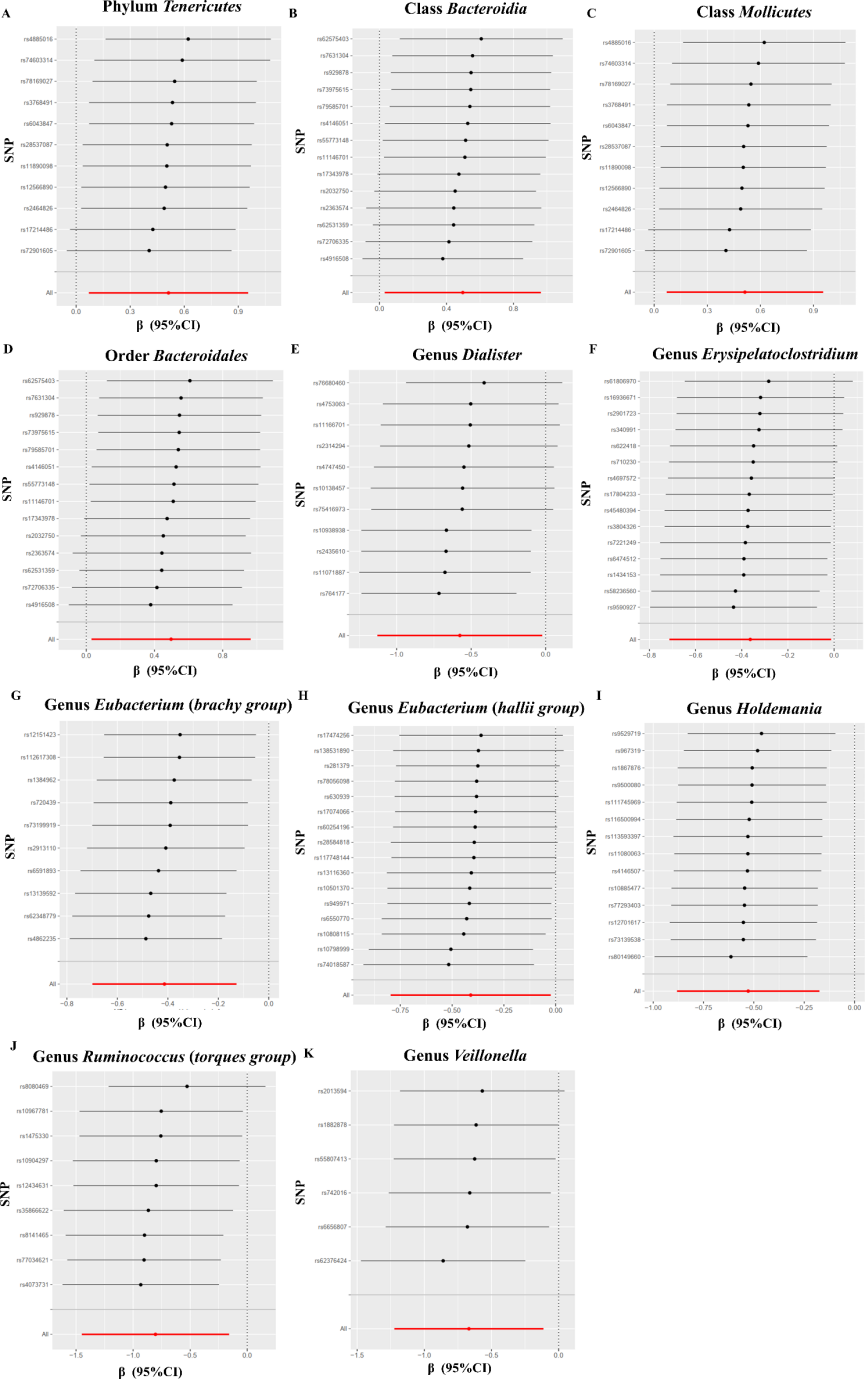
Leave-one-out plots for the causal effect of gut microbiota on ICP. **(A)** Phylum *Tenericutes***(B)** Class Bacteroidia **(C)** Class *Mollicutes***(D)** Order *Bacteroidales***(E)** Genus *Dialister***(F)** Genus *Erysipelatoclostridium***(G)** Genus *Eubacterium* (*brachy group*) **(H)** Genus *Eubacterium* (*hallii group*) **(I)** Genus *Holdemania***(J)** Genus *Ruminococcus* (*torques group*) **(K)** Genus *Veillonella*

Cochran’s IVW Q test showed that there was no substantial heterogeneity among the chosen IVs (Table S2). There was no evidence of horizontal pleiotropy according to the results of the MR-Egger intercept analysis (Table S3). No pleiotropic SNP was found in the analysis of class *Bacteroidia* (*P* = 0.529), order *Bacteroidales* (*P* = 0.541), genus *Dialister* (*P* = 0.183), genus *Erysipelatoclostridium* (*P* = 0.804), genus *Eubacterium* (*brachy group*) (*P* = 0.523), genus *Eubacterium* (*hallii group*) (*P* = 0.715), genus *Holdemania* (*P* = 0.958), genus *Ruminococcus* (*torques group*) (*P* = 0.447), and genus *Veillonella* (*P* = 0.789) assessed by MRPRESSO analysis. We identified, rs10108398 as outlier for the association between phylum *Tenericutes* and ICP risk. Besides, the results of MR-PRESSO global test showed that there was significant horizontal pleiotropy between the IVs associated with class *Mollicutes* (*P* = 0.002), and we identified rs10108398 an outlier SNP. After removal of these pleiotropic SNPs, the outlier-corrected results showed that there is no evidence of horizontal pleiotropy of the remaining IVs (Table S3). In the leave-out sensitivity analysis, we discovered that none of the risk estimates between particular bacterial taxa and risk of ICP were caused by a single SNP (Fig. 3).

### Reverse MR analysis

We performed a reverse MR analysis to infer whether there was a causal link between ICP and relative abundance of 11 bacterial taxa. We selected the SNPs (*P* < 5 × 10^− 8^) significantly associated with the risk of ICP as IVs (Table S4). The results indicated there was no reverse causal link between ICP and identified bacterial features (Table S5). Q statistics of the IVW test demonstrated no significant heterogeneity across the included IVs (Table S6). No substantial horizontal pleiotropy was identified in the MR-Egger intercept analysis and MR-PRESSO global test (Table S7). In the leave-out sensitivity analysis, strongly affecting SNPs were identified in the IVs of genus *Erysipelatoclostridium* (rs17209837), genus *Eubacterium* (*hallii_group*) (rs17209837), and genus *Veillonella* (rs1260326). After excluding the one, substantially influential IV, the results were still valid (Fig. S1).

## Discussion

To the best of our knowledge, this study is the first to use published available GWAS summary statistics to undertake a bidirectional two-sample MR analysis to investigate the causal associations between gut microbiota and ICP risk. We found that ICP risk was associated with a higher abundance of the phylum *Tenericutes*, class *Bacteroidia*, class *Mollicutes*, and order *Bacteroidale*, and. By contrast, we observed that higher levels of the genus *Dialister*, genus *Erysipelatoclostridium*, genus *Eubacterium* (*brachy group*), genus *Eubacterium* (*hallii group*), genus *Holdemania*, genus *Ruminococcus* (*torques group*), and genus *Veillonella* were related to a lower risk of ICP.

Although the precise biological mechanism underlying ICP’s occurrence is still unknown, earlier observational studies have suggested that gut microbiota played a significant part in the disease’s etiology. A case-control study performed by Li et al. showed that the butyrate-producing bacteria including genus *Eubacterium* (*hallii group*), were depleted in ICP patients, which was in line with our results [26]. According to Zhan et al., the severe ICP group displayed considerable gut microbiota dysbiosis and had increased microbial gene functions for propanoate metabolism and the manufacture of unsaturated fatty acids [27]. However, there was no consensus on whether there was a causal effect of gut microbiota on the occurrence of ICP. In this study, a two-sample MR analysis successfully identified that phylum *Tenericutes*, class *Bacteroidia*, and order *Bacteroidale* were positively associated with ICP risk. The phylum *Bacteroidetes* possess the enzyme bile salt hydrolase which could deconjugate bile acids, reduce ileal bile acid uptake, decrease the induction of FXR, and reduce the level of fibroblast growth factor 19/15 (FGF19/15), thereby increasing synthesis of bile acid in the liver [28, 29]. Tang et al. found that a notable difference in microbial profiles between ICP cases and healthy controls, and the microbiomes of patients with ICP were primarily characterized by *Bacteriodes fragilis*, which was consistent with our findings [30]. The phylum *Tenericutes* are positively associated with pro-inflammatory factors IL-6, TNF-α, and IL-17 A, which were significantly increased in ICP patients [31, 32]. Furthermore, we proved some bacterial taxa were negatively associated with ICP risk. Genus *Dialister* [33], genus *Eryipelatoclostridium* [34], genus *Eubacterium* [35], genus *Ruminococcus* [36] produce butyrate, which plays an important role in energy homeostasis, maintenance of the gut barrier functions, immunomodulation, and anti-inflammation [37]. The genus *Eubacterium* could also regulate the expression of several enzymes during the metabolism of bile acids, including, 7α-hydroxylase (Cyp7a1), oxysterol 7α-hydroxylase (Cyp7b1), and sterol 27-hydroxylase (Cyp27a1), and to determine the amount of bile acids produced [38]. We also discovered that class *Mollicutes*, genus *Holdemania*, and genus *Veillonella* were associated with ICP. However, there have not been many prior investigations on how these bacteria affect ICP etiology. A theoretical foundation for the use of probiotics to treat ICP should be provided by investigations on the molecular mechanisms underlying these gut microbiota compositions in ICP.

This research has several strengths. First, MR analysis is unlikely to be influenced by the interference of confounding factors and reverse causality compared with traditional observational study design. In addition, we collected the genetic variants from the largest available GWAS meta-analysis for human gut microbiota composition, ensuring the strength of genetic instruments in this MR analysis. To ensure that our results are not the product of pleiotropic effects, we also employed the MR-Egger intercept analysis and MR-PRESSO method to examine for any potential horizontal pleiotropy on the observed causal correlations.

Still, our analysis has several potential limitations. First, the present study included only participants of European ancestry, which minimize the risk of confounding due to population admixture but may limit the generalizability of our findings to different populations. Second, the two-sample MR design only tested the linear effect of the relative abundance of gut microbiota composition on ICP risk in the general population. Third, The GWAS summary statistics for gut microbiota were not restricted to the female population [16]. Although the genetic variants located on the sex chromosomes were excluded, as well as sex was adjusted in the analysis [16], the potential bias due to sex could not be totally excluded. Fourth, the precise biological mechanisms of the influence of specific intestinal flora on the pathogenesis of ICP were still unclear. To better understand the impact of gut microbiota on ICP, additional clinical and functional research was required.

## Conclusions

In summary, we comprehensively assess the potential causal relationship between gut microbiota and ICP. This two-sample MR study provide precise evidence that the relative abundance of several gut microbiota was causally associated with ICP. To better understand how probiotics affect ICP and its precise biological mechanism, additional research that incorporates the findings of well-designed randomized controlled trials is necessary. Additionally, although reverse MR analysis did not support the causal effect of ICP on gut microbiota, it cannot be ruled out that ICP may influence intestinal microorganisms, which needs to be verified by further research.

### Electronic supplementary material

Below is the link to the electronic supplementary material.

Supplementary Material 1

Supplementary Material 2

## Data Availability

The datasets analyzed in this current study can be accessed through the following links: microbiota, https://mibiogen.gcc.rug.nl/; intrahepatic cholestasis of pregnancy, https://r5.finngen.fi/pheno/O15_ICP.

## Abbreviations

GWAS: Genome-wide association study
MR: Mendelian randomization
SNP: Single nucleotide polymorphism
IV: Instrumental variable
IVW: Inverse variance weighted
ML: Maximum likelihood
MR-PRESSO: Mendelian randomization pleiotropy residual sum and outlier
LD: Linkage disequilibrium
ICP: Intrahepatic cholestasis of pregnancy
OR: Odds ration
CI: Confidence interval
SCFA: Short-chain fatty acid
