# Supplementary material for: Causal association between gut microbiota and intrahepatic cholestasis of pregnancy: mendelian randomization study
Source: BMC Pregnancy Childbirth. 2023 Aug 5;23:568. doi: 10.1186/s12884-023-05889-8 (PMC10403878; doi:10.1186/s12884-023-05889-8)
Supplement: Supplementary file 2 — Supplementary Material 2 [file 12884_2023_5889_MOESM2_ESM.docx]

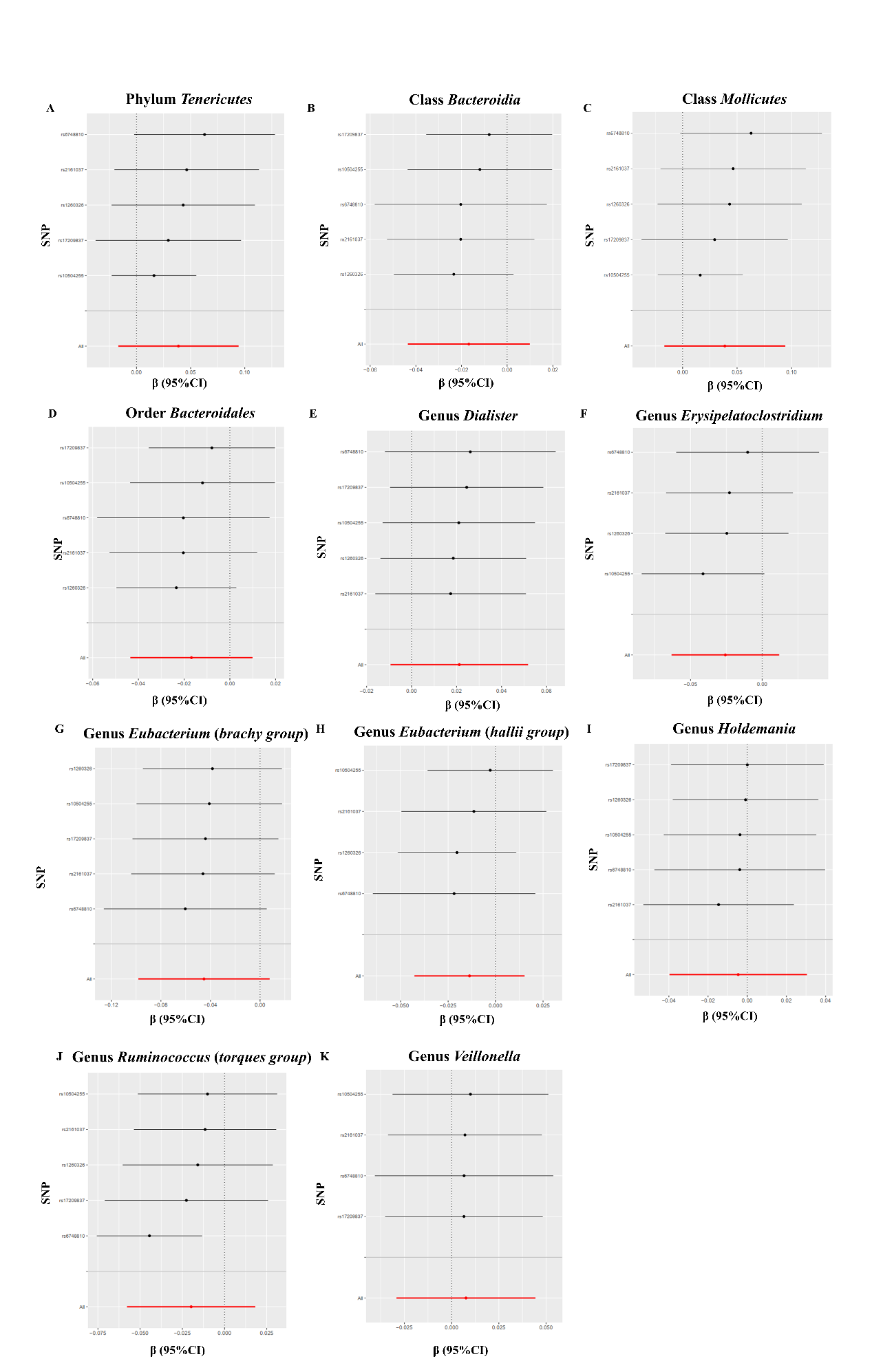


**Additional file 2. Supplementary figure. Fig. S1. Leave-one-out plots for the causal effect of ICP on gut microbiota.** **(A)** Phylum *Tenericutes* **(B)** Class Bacteroidia **(C)** Class *Mollicutes* **(D)** Order *Bacteroidales* **(E)** Genus *Dialister* **(F)** Genus *Erysipelatoclostridium* **(G)** Genus *Eubacterium* (*brachy group*) **(H)** Genus *Eubacterium* (*hallii group*) **(I)** Genus *Holdemania* **(J)** Genus *Ruminococcus* (*torques group*) **(K)** Genus *Veillonella*
